# Cell Distribution within Yeast Colonies and Colony Biofilms: How Structure Develops

**DOI:** 10.3390/ijms21113873

**Published:** 2020-05-29

**Authors:** Vítězslav Plocek, Libuše Váchová, Vratislav Šťovíček, Zdena Palková

**Affiliations:** 1 Department of Genetics and Microbiology, Faculty of Science, Charles University, BIOCEV, 12800 Prague, Czech Republic; vitezslav.plocek@natur.cuni.cz (V.P.); v.stovicek@seznam.cz (V.Š.); 2 Institute of Microbiology of the Czech Academy of Sciences, BIOCEV, 14220 Prague, Czech Republic; vachova@biomed.cas.cz

**Keywords:** yeast multicellular structures, colonies and biofilms, structure development, cell organization, laboratory and wild *Saccharomyces cerevisiae* strains, cell adhesion, Flo11p adhesin

## Abstract

Multicellular structures formed by yeasts and other microbes are valuable models for investigating the processes of cell–cell interaction and pattern formation, as well as cell signaling and differentiation. These processes are essential for the organization and development of diverse microbial communities that are important in everyday life. Two major types of multicellular structures are formed by yeast *Saccharomyces cerevisiae* on semisolid agar. These are colonies formed by laboratory or domesticated strains and structured colony biofilms formed by wild strains. These structures differ in spatiotemporal organization and cellular differentiation. Using state-of-the-art microscopy and mutant analysis, we investigated the distribution of cells within colonies and colony biofilms and the involvement of specific processes therein. We show that prominent differences between colony and biofilm structure are determined during early stages of development and are associated with the different distribution of growing cells. Two distinct cell distribution patterns were identified—the zebra-type and the leopard-type, which are genetically determined. The role of Flo11p in cell adhesion and extracellular matrix production is essential for leopard-type distribution, because *FLO11* deletion triggers the switch to zebra-type cell distribution. However, both types of cell organization are independent of cell budding polarity and cell separation as determined using respective mutants.

## 1. Introduction

In most natural environments, microbes occur in the form of structured populations such as biofilms and other types of microbial consortia. Whether biofilms of commensal or potentially pathogenic microbes, microbial consortia that decompose waste products, or populations used in the food industry, all of these microbial communities significantly affect the lives of other organisms (including human). Understanding the relationships among microbes in such populations is the first step toward regulating their development and, where necessary, defending against them. 

Yeast, similar to other microbes, form various types of multicellular communities that differ in the complexity of their organization. These include diverse types of colonies, biofilms, or mats grown on solid/semisolid surfaces, flor biofilms at the borders between liquid and air environments, and flocs composed of aggregated cells in liquid environments [1,2,3,4,5,6,7,8,9,10]. Yeast cells that are differently positioned within these structures differ in their ability to access nutrients and gases (namely oxygen), to remove waste products (including CO_2_), and to interact with neighboring cells. As a consequence, cells at different positions within the structure acquire distinct properties, i.e., start to differentiate to form different cell types. Then, differentiated cells enhance the heterogeneity of the structured environment, which in turn contributes to further stages of cell diversification due to ambient conditions, such as gradients of metabolites and signaling molecules produced by adjacent cells. Those multicellular structures that exhibit high levels of three-dimensional organization (such as colonies and colony biofilms) also exhibit complicated internal organization. 

Two major types of structures are formed by yeast *Saccharomyces cerevisiae* grown on semisolid agar. These are smooth colonies formed by most laboratory strains as well as by strains derived by domestication from wild strains and structured colony biofilms formed by some wild strains. Both colonies and colony biofilms are formed by the division of non-motile yeast cells. These cells pass through various stages of differentiation, which are related or unrelated to division and dependent on the growth conditions and properties (e.g., genetic background) of a particular strain. As a result, the three-dimensional (3D) architecture of smooth colonies and colony biofilms differs dramatically [3,8]. 

Smooth colonies can arise either from single cells (microcolonies) or from cell suspensions of genetically identical cells (giant colonies). Independently of the initial number of inoculated non-differentiated cells, from a specific point of colony growth, further cell development in colonies is coordinated, and cell differentiation is guided by a specific developmental program [11,12]. On complex, respiratory agar medium, *S. cerevisiae* smooth colonies undergo development characterized by phases of acidification and alkalization. After a short initial alkalization (approximately 24 h), giant colonies enter an acidification phase lasting approximately 8–9 days, during which colonies grow linearly. This is followed by the initiation of alkalization that is associated with the production of volatile ammonia, which functions as a signal that is involved in colony synchronization and cell differentiation [8,13,14]. The development of microcolonies is faster and depends on the density of colonies in a territory; the higher the number of colonies, the faster their development [12]. Transition from the acidic phase to the alkali, ammonia signaling period is a key point in colony development, as it is associated with colony differentiation to two major cell types that are specifically positioned within the structure [11,12]. These are U cells localized to upper regions and L cells in internal/lower colony areas. U and L cells significantly differ in morphology, metabolism, and longevity [11], and they gain various features that become apparent only as colonies differentiate upon ammonia signaling. For example, U cells display an unusual combination of metabolic and regulatory features, some of which typically occur in stationary phase yeast cells in liquid cultures, some occur in metabolically active growing cells, and others are specific to colony U cells. Starving L cells are in a resting state and activate various hydrolytic pathways [8,11].

Architecture and properties of *S. cerevisiae* colony biofilms (formed either from single cells or from cell suspension) differ significantly from those of smooth colonies [15,16]. The colony biofilm structure is strongly affected by nutrients, being highly wrinkled on respiratory medium, but less wrinkled or even rather smooth on high glucose media. When glucose is spent, colony biofilms start to acquire structured morphology [17]. Therefore, the most prominent architectural differences between smooth colonies and colony biofilms appear on respiratory media. On such media, colony biofilms are composed of two major parts, one formed mostly of oval shaped cells above the agar (the aerial part) and one inside the agar (biofilm “roots”) composed mostly of pseudohyphae invading the agar [16,18]. Cell wall adhesin Flo11p and its regulators Cyc8p and Tup1p are essential for colony biofilm formation [5,18,19,20]. Different types of cell differentiation have been identified in colony biofilms, some of which contribute to numerous strategies that help to protect the structure against the environment [16]. Surface cell layers are protected via active plasma membrane multidrug resistance transporters Pdr5p and Snq2p, which expel toxic compounds. Cells in the biofilm interior are embedded in the extracellular matrix (ECM), which is believed to have a dual function as a nutrient reservoir and as a low-permeability barrier, blocking the penetration of some compounds (including toxic ones) [16]. 

Architectural differences and the presence of different cell types in smooth colonies vs. colony biofilms raise an important question: how is the early organization of cells reflected in the resulting differentiated structure? Specifically, whether (and how) the early behavior of laboratory (domesticated) strains and wild strains differ, and whether such differences are reflected in different modes of cell organization and pattern formation. We show here that cells are distributed differently from the beginning of architecture development of smooth colonies and colony biofilms. Furthermore, we show that cell distribution is independent of budding polarity and cell separation, but it is strongly dependent on cell–cell attachment via adhesins and ECM from the early stages of colony formation. 

## 2. Results

### 2.1. Dynamics of Cell Distribution Differ in Smooth Colonies and Colony Biofilms

**Figure 1:**
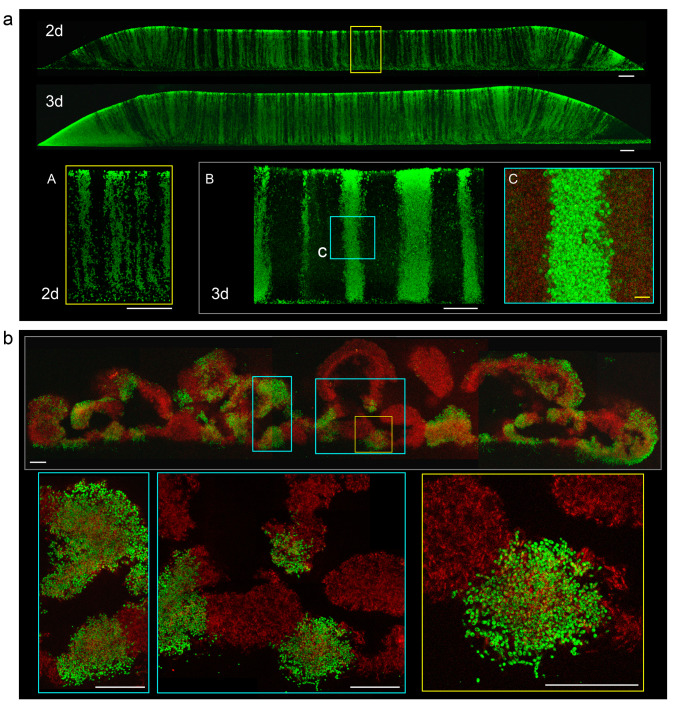
Vertical cross-sections of chimeric smooth colonies and colony biofilms. (**a**) Cross-sections visualized by two photon confocal microscopy (2PE-CM) of 2-day-old and 3-day-old chimeric giant colonies of strains BY4742 (black stripes) and BY-p_TEF_-GFP (green stripes), mixed equally, and inoculated as 1 μL drops of 10^8^ cells/mL. Lower panel, details of stripes in higher magnification, of 2-day-old colony as above (A) and from 2-day-old colony inoculated by 1 μL drops of 10^6^ cells/mL (B and inset C). White bar, 100 μm, yellow bar, 10 μm. (**b**) Cross-sections of chimeric giant colony biofilm formed by biofilm-forming cells (BR-F) and BR-F-p_TEF_-GFP mixed equally and inoculated as 1 μL drops of 10^8^ cells/mL. Here, the autofluorescence of “black” BR-F cells is visualized as a false red color. The cell-free areas filled with extracellular matrix (ECM) are black. Insets show parts of the colony biofilm in higher magnification. Bar, 100 μm.

To distinguish areas settled by particular cells and thus monitor cell distribution within smooth colonies and colony biofilms from the early phases of their development, we constructed strains (Table 1) derived from BY4742 (laboratory strain) and BR-F (wild strain), expressing gene for green fluorescent protein (GFP) from the constitutive p_TEF_ promoter, which ensures almost the constitutive expression of GFP within colonies [12]. Then, we grew chimeric giant colonies formed from a suspension of smooth colony-forming cells (BY4742 mixed with BY-p_TEF_-GFP) or biofilm-forming cells (BR-F mixed with BR-F-p_TEF_-GFP), both mixed in different ratios and plated at different densities on respiratory medium GMA. Using cells of two colors—green (expressing GFP) and black (not expressing GFP; visualized in some experiments using autofluorescence measurement)—allowed us to study the distribution of cells derived from either black or green cell ancestors. Patterns of black and green cells in developing giant colonies and biofilms were analyzed using colony cross-sectioning and two photon confocal microscopy (2PE-CM) (Figure 1).

Chimeric smooth colonies were inoculated using suspension containing equal numbers of green and black cells at concentrations of about 10^8^ cells/mL and vertical cross sections of 3-day-old colonies analyzed by 2PE-CM. The regular vertical distribution of green and black cells was observed in central parts of the colony roughly corresponding to the site of inoculation, whereas a diagonal pattern of cells was observed at margin regions (Figure 1a). This “zebra”- type patterning was independent of the ratio of inoculated strains and density of plating; cell density influenced only the thickness and number of black and green cell columns, appearing as zebra stripes in cross-sections. In colonies inoculated with a lower cell number (lower cell density of the inoculum), the black and green columns were thicker than in colonies inoculated with a higher cell number (higher cell density) (Figure 1a). The relative number and thickness of green and black columns also depended on the ratio of green to black cells in the inoculation suspension. The vertical pattern of green and black cells was still clearly visible in later phases of colony development in upper cell layers differentiated to U cells. Hence, the process of U and L cell differentiation is independent of primary distribution of cells within smooth colonies. 

Chimeric colony biofilms were grown on GMA from a spot inoculated with a mixture of equal numbers of BR-F and BR-F-p_TEF_-GFP cells. Vertical cross-sections of 3-day-old biofilms were analyzed by 2PE-CM; black cells were detected by autofluorescence for easier orientation in the structure (Figure 1b; false red color). In contrast to the zebra phenotype of smooth chimeric colonies, the pattern of green and black cells in chimeric colony biofilms was irregular. The chimeric biofilms were composed of larger and smaller red and green cell areas spread within the biofilm rather randomly (Figure 1b). The black areas present within green/red areas are cell-free cavities containing ECM. This “leopard-type pattern was independent of plating density.

### 2.2. “Zebra” Versus “Leopard” Cell Distribution Arises in Early Stages of Colony/Biofilm Development

**Figure 2:**
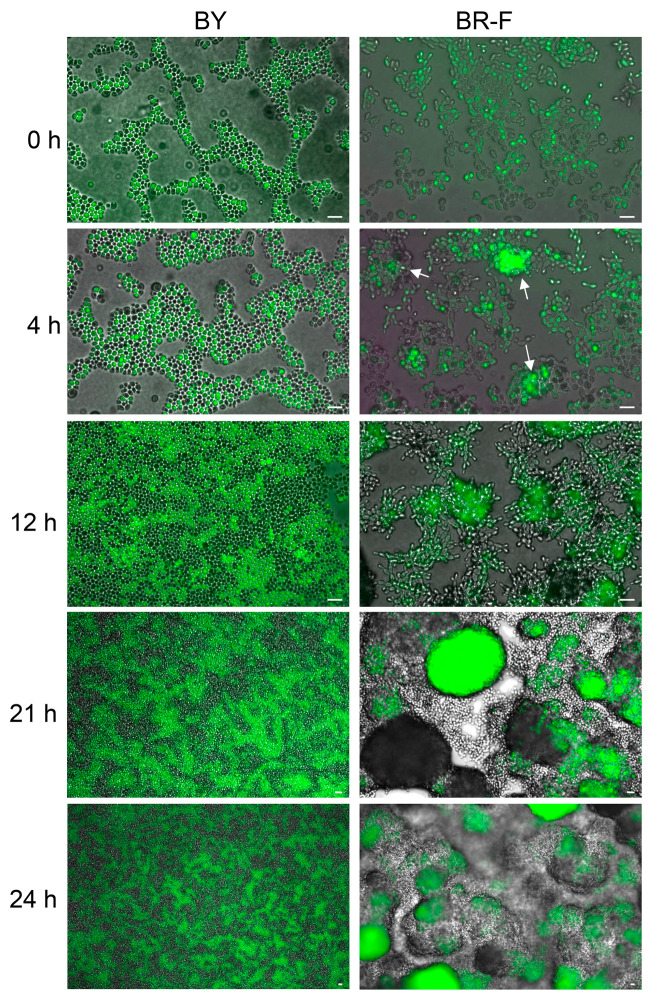
Timeline of cell growth in inoculated area. Agar surface was inoculated with a mixture of BY4742 (black cells) and BY-p_TEF_-GFP (green cell), and with a mixture of BR-F (black cells) and BR-F-p_TEF_-GFP (green cell), the strains were mixed equally as in Figure 1; 1 μL drops of 10^8^ cells/mL were used for inoculation. The presence of black and green cells was detected by combining GFP fluorescence imaging and bright field microscopy. Arrows indicate examples of BR-F/BR-F-p_TEF_-GFP cell clusters already forming 3D structures after 4 h of growth.

Next, we analyzed cell growth during the initial interval of 0–24 h after the inoculation of both types of chimeric structures. Black and green cells were inoculated with equal number of cells at standard density (Figure 2, time 0 h). During the initial approximately 12 h, BY-derived cells grew horizontally on the agar surface, mostly as a monolayer, forming mosaic structures of black and green cells (Figure 2, time 12 h), which remained clearly visible in later stages when viewed from above (Figure 2, time 21 and 24 h). Thereafter, cells started to grow vertically and form 3D structure. Since the growth rates of green and black cells were similar, each cell type could expand only into the vertical space above the agar area, which was occupied by that particular cell type during the initial horizontal growth. As a result, relatively regular green and black columns were formed (Figure 1a). In contrast, BR-F-derived cells, from the outset, started to create small 3D structures, already visible after 4 h of growth (Figure 2, 4 h, examples indicated by arrows). Thus, individual 3D microcolonies are formed much earlier, before the chimeric cell population has covered the surface of the inoculated agar area (between 21 and 24 h), and they become larger as the chimeric colony grows and apparently form the basis of the leopard-type structure of the biofilm. 

**Figure 3:**
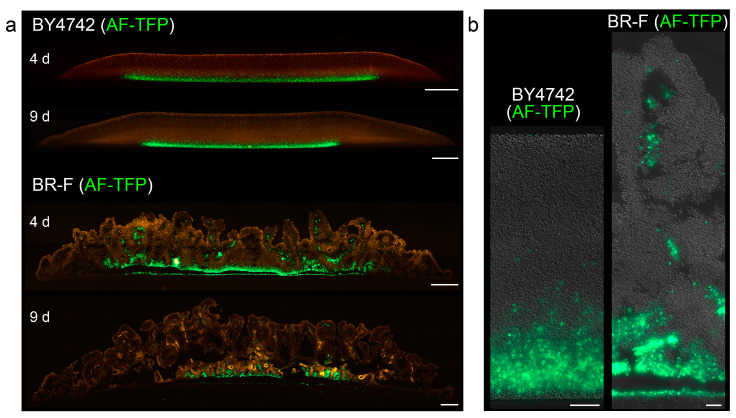
Distribution of cells stained with AlexaFluor488 5-TFP in giant colonies and colony biofilms. Cross-sections of whole 4 and 9-day-old smooth colonies (BY4742) and colony biofilms (BR-F) (**a**) and parts of the structures in higher magnification (**b**). In green, cells stained with AlexaFluor488 5-TFP (AF-TFP) used for inoculation. Offspring cells are indicated in false red: autofluorescence (a) and in differential interference contrast (DIC) (b) Bar, 500 μm (a), 50 μm (b).

Next, we analyzed the 3D distribution of originally inoculated cells and offspring cells in both types of structure. We vitally stained the proteins on the surface of BY4742 and BR-F cells with AlexaFluor488 5-TFP (AlexaFluor488 carboxylic acid, 2,3,5,6-tetrafluorophenyl ester amine-reactive probe) and used them for colony/biofilm inoculation. During yeast cell division, the cell wall material of the mother cells is not redistributed to the daughters, and thus stained proteins remain attached to the originally labeled cells [21,22]. A relatively high number of stained cells was used for the inoculation of giant colonies/biofilms to be able to monitor the localization of original stained cells, using colony cross-sections and fluorescence microscopy. Stained cells remained localized to the lower parts of smooth colonies (Figure 3), which, together with the zebra pattern observed in chimeric colonies, indicated that offspring were mostly distributed upwards in a vertical direction. Only occasionally, a few stained cells were re-located to higher colony areas. From day 3–4, an additional thin layer of unstained cells appeared at the colony bottom, indicating that a few cell divisions also occurred in this region. In BR-F biofilms, the majority of stained cells also remained in lower regions. However, a significant number of stained cells (and even clusters of these cells) was relocated upwards, relatively far from the site of their inoculation, and they became localized mostly within the wrinkles even in older colonies, some of the clusters reaching almost to the surface of the aerial part (Figure 3). 

### 2.3. Zebra vs. Leopard Cell Distribution Depends on Cell Adhesiveness, but Not on the Type of Cell Division or Cell Separation

BY4742 and BR-F structures differ in various key characteristics including cell adhesiveness related to the formation of cell clusters, the production of the extracellular matrix, and a dimorphic switch, related to an ability to form pseudohyphae [8]. Some of these features are dependent on *FLO11*, which is the gene for Flo11p surface adhesin, the production of which is reduced in smooth colonies [18]. Haploid and diploid yeast strains differ also in budding polarity [23,24], which is axial/random in haploid BY4742 and unipolar/bipolar/random in BR-F [18]. Therefore, we analyzed, which of these parameters, if any, could be the key factor in zebra vs. leopard cell distribution and thus in the organization of the colonies/biofilms. We constructed a series of strains derived from BY4742/BY4742-p_TEF_-GFP and/or BR-F/BR-F-p_TEF_-GFP, which are deleted in genes involved in the above-mentioned characteristics (Table 1). Specifically, we constructed knockout (KO) randomly budding strains *bud2* and *rsr1* deleted in genes involved in bud-site selection [23], strain *ace2* with cell separation defects [25], and strain *flo11*. Respective pairs of KO strains (labeled and non-labeled with GFP) were used for the inoculation of chimeric colonies/biofilms. 

**Figure 4:**
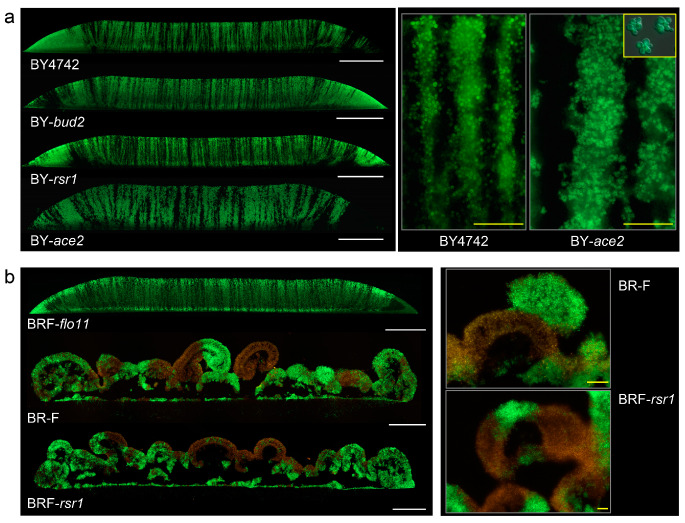
The effect of impaired budding polarity, cell separation, and cell adhesion on the formation of chimeric giant colonies and colony biofilms. (**a**) Cross-sections of chimeric colonies formed by knockout (KO) strains as indicated, derived from BY4742; the BY4742 colony is shown as a control. Details of BY-*ace2* stripes as compared to BY4742 stripes are shown in the right panel. Inset (yellow rectangle), individual clusters of non-separated BY-*ace2* cells grown in GM liquid medium. White bar, 500 μm; yellow bar, 50 μm. (**b**) Whole cross-sections of chimeric colony biofilm formed by KO strains in gene *RSR1* or *FLO11* derived from BR-F; BR-F colony biofilm as a control (white bar, 500 μm), and examples of aerial parts in higher magnification (right; yellow bar, 100 μm). 1 μL drops of 10^8^ cells/mL were used for inoculation.

The deletion of either *BUD2* or *RSR1* genes leading to the random budding of BY4242 did not affect zebra-type cell distribution (Figure 4a). As expected, the deletion of *ACE2* caused defects in cell separation, and thus clusters of non-separated cells were formed in BY-*ace2* culture (Figure 4a), partially resembling cell clusters observed in colony biofilms [16,26]. However, the formation of these cell clusters did not cause a prominent change in colony structure and zebra-like cell distribution. The only difference was that the stripes were thicker and the edges of the stripes were more frayed than in BY4742 colonies (plated in the same density) due to the presence of the clusters (Figure 4a, right panel). Hence, defects in neither cell budding polarity nor cell-cluster formation cause a change from zebra to leopard cell distribution. 

Deletion of the *RSR1* gene in strain BR-F did not affect the formation of colony biofilm and leopard-type cell distribution (Figure 4b). However, deletion of the *FLO11* gene, which disrupts both cell adhesion and ECM production and leads to smooth colony formation [18,20,26], completely changed cell distribution from leopard to zebra type (Figure 4b). Hence, it is the ability of cells to adhere, and maybe to produce ECM, not budding polarity, that determines leopard-type cell distribution. 

## 3. Discussion

**Figure 5:**
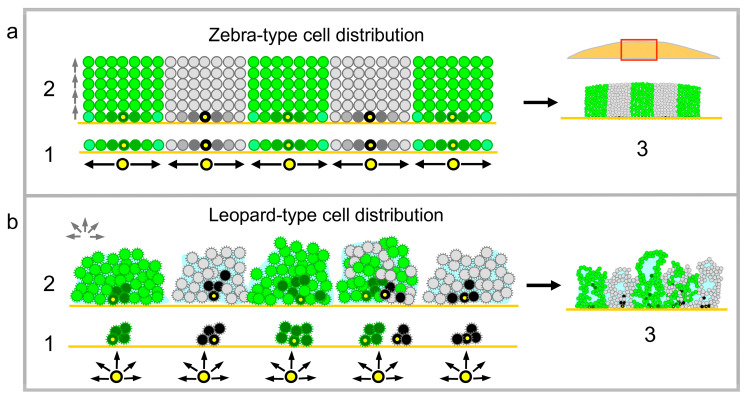
Model of cell distribution during the formation of colonies (**a**) and colony biofilms (**b**). Schematic view on cross-sections of young chimeric giant colony (about 12 h old) or colony biofilm (about 4-h-old) (1); 24-h-old (2); and 3-day-old (3) structures. Black and gray cells show old and younger black cells, respectively, dark and light green show old and younger green cells, respectively. For 1 in a, the lighter the color of the cell, the younger the cell. Original inoculated cells are marked in yellow. Red rectangle in a/3 shows the localization of green/black schematic within the whole colony cross-section. Black and green arrows indicate the direction of cell growth in 1 and in 2; light blue, ECM; cell surface brushes in (**b**), adhesive fibers.

According to their properties, yeast strains form either colony biofilms under specific nutritive conditions such as respiratory media or smooth colonies under any circumstances. Wild strains can switch off their ability to form biofilms (and all biofilm-specific features) after sustained growth on plentiful nutrients such as high glucose [15,27]. This process, called domestication, is reversible, as strains are able to switch back to biofilm mode under severe stress and starvation conditions [27]. In contrast, laboratory strains, which were domesticated centuries ago and adapted to high nutrients over a long period, always form smooth colonies independently of conditions of their growth. The smooth-colony and structured-biofilm lifestyles differ in many parameters, including the organization of architecture of and cell differentiation in these structures [8]. We show here that the internal organization of the smooth-colony vs. structured-biofilm population is determined in the early phases of structure development and results in two fundamentally different modes of cell distribution: “zebra-type” and “leopard-type” (Figure 5), each exhibiting specific characteristics.

In zebra-type chimeric giant colonies, growing cells first occupy the inoculation area, forming a cell monolayer. Thereafter, when no “horizontal” space on the agar is available, cells are forced to grow upwards in a vertical direction. As the growth rate of green and black strains is the same, the green/black offspring cells can only fill the space above the agar area that each type initially occupied, thus creating relatively regular columns of cells seen as stripes in vertical colony cross-sections (Figure 5a). Hence, in this type of structure, cells that are chronologically older are present in lower areas of colonies (near to the bottom), whereas only a few older cells are occasionally pushed upwards to reach higher layers of the colony. The younger offspring cells are distributed upwards to areas, which are further from the nutrients in the agar. The thickness of the columns of cells originating from one cell ancestor plated during giant colony inoculation depends on the density of cell plating, which defines a space around each ancestor cell that can subsequently be occupied by its progeny during the monolayer growth phase (Figure 5a). Differentiation to U and L cells, accomplished later as the colonies become aged and undergo ammonia signaling [11], was identical in green and black columns of chimeric giant colonies composed of cells that are genetically identical with the exception of GFP. Thus, the formation of U and L cells is dependent on the particular cell position within the colony and the stage of colony development, but not on cell history. For example, U cells were always formed in upper cell layers, in which the cells are the progeny of different vertically grown ancestor cells (Figure 1a).

In leopard-type giant colony biofilms, cell distribution is less regular than in zebra structures. From the first hours of development, cells remain attached to each other and to the semisolid agar and form small three-dimensional microcolonies (Figure 5b). These microcolonies are either composed of the progeny of a single-type ancestor (containing only black or green cells in chimeric biofilms) or several ancestors that have been inoculated in close proximity and are thus attached to each other (microcolonies composed of both green and black cells). The microcolonies continue to grow in all 3 dimensions and only later join to form one giant colony biofilm, which includes various aerial wrinkles. Individual wrinkles can either all be black or green, or they can be a mosaic structure (black and green), which is probably dependently on whether they arise from microcolonies of single-type ancestors or those of both types of ancestors. The production of ECM, which apparently participates in the formation of wrinkles, could contribute to the relatively irregular positioning of black and green cell clusters within the leopard structures. As shown in colony biofilms grown from AlexaFluor488 5-TFP stained cells, a significant number of the stained cells is distributed upwards to different colony wrinkles (far from the inoculated layer of stained cells, which remain at the bottom of the biofilm). Hence, in contrast to zebra-type structures, chronologically older and younger cells can be more mixed in leopard-type biofilms, due to cell–cell adhesion and mechanical forces of the ECM. On other hand, in leopard-type biofilms, as in zebra structures, cell differentiation leading to specific spatially positioned cell types such as cells with active multidrug resistance transporters or cells producing ECM [16] does not correlate with the pattern of cell distribution in the biofilm, but it does correlate with the cell position in the structure. 

Both zebra-type and leopard-type cell distributions are independent of the genetically controlled polarity of cell division as shown using knockout strains (*bud2*, *rsr1*) with disrupted bud site selection. This finding is in agreement with previously published data showing that changes in cell polarity and budding influence morphology of yeast colonies only moderately or not at all [28]. In contrast to BY4742 colonies, in which cells do not mutually adhere despite being close to one another, BR-F cells are usually present in clusters, even after they are separated from colony biofilms or grown in liquid cultures. Flo11p adhesin is essential for many processes related to biofilm development (including cell adhesion, the formation of thin fibers connecting cells within biofilms, and ECM production) [16,18,19,20,26]; it is present in cell–cell adhesion sites of cell clusters formed by strain Σ1267 and in higher concentrations than in other areas of the cell wall [26]. Strain Σ1267 forms colony biofilms similar to those of BR-F [16,26]. A defect in cell separation during division due to the deletion of the *ACE2* gene causes cell clustering of BY4742 cells, which at first glance is similar to the clustering of BR-F cells. However, the mechanisms of formation of these two types of clusters are different. BY-*ace2* clusters are formed via incomplete cell separation during division where daughter cells remain associated with their mothers [25], whereas BR-F clusters are formed via the adhesion of cell walls of already separated cells, thus potentially of daughters of different mothers. Cluster formation by incomplete cell separation in BY-*ace2* did not change zebra-type colonies to leopard-type biofilms, whereas deletion of the *FLO11* adhesin gene in BR-F switched leopard-type cell distribution to zebra-type cell distribution completely. Hence, a simple ability to form cell clusters is not sufficient to switch from zebra-to-leopard cell distribution, but the ability of cells to mutually adhere (independently of how they are related) due to their cell wall properties is a key parameter for this switch. Possibly, in contrast to BY-*ace2* clusters that are rather rigid, cell-wall/fiber-mediated adhesion is more flexible and allows the reorganization of cell groups during biofilm development, as indicated also by the mosaic pattern of black and green cell clusters in chimeric colonies. Thus, the relationship among some of the adhered cells in colony biofilms could resemble that of cells in flocs, pellicles, and flor biofilms [7,10], which are formed by the adhesion of cells that are not directly related. 

In summary, new findings indicate that properties related to cell wall, adhesins, and ECM are key parameters that can distinguish the ability of cells to organize differently and subsequently to form different types of multicellular structures on semisolid media. This contrasts with findings that identified defects in cell division (for example due to *ACE2* deletion) and the formation of so-called snowflake yeast as potential mechanisms involved in the origins of multicellularity in liquid cultures [29]. 

## 4. Materials and Methods 

### 4.1. Yeast Strains and Cultivation

All *S. cerevisiae* strains prepared in this study (Table 1) were derived from the wild strain BR-F from a collection at the Institute of Chemistry (Slovak Academy of Sciences) or from the laboratory strain BY4742 from the Euroscarf collection. Giant colonies were inoculated with 1 μL of cell suspension (concentration 10 mg wet weight biomass/mL corresponding to 10^8^ cells/mL, approximately, unless otherwise indicated) and grown on respiratory GMA medium (3% glycerol, 1% yeast extract, and 2% agar) at 28 °C. Biofilm structures are formed on respiratory medium, whereas biofilm formation is inhibited on glucose medium [9,17]. GM liquid medium (GMA without agar) was used in some experiments.

Chimeric colonies were inoculated with a mixed suspension composed of an equal number of cells of two strains unless otherwise stated. Representative experiments of at least three independent replicates are shown. 

### 4.2. Strain Constructions

Strains expressing GFP under the control of constitutive promoter p_TEF_ were constructed using integration cassettes amplified from plasmids pYM-N21 (nat selection; from Euroscarf collection) and pHLA21 (derived from pYM-N21 by including hph selection gene; provided by O. Hlaváček) integrated into the *HIS3* locus of BY4742 and BR-F strains according to [30]. Gene knockouts were performed by transforming the cells with deletion cassettes generated by PCR from plasmid pUG6 (kanMX selection, from Euroscarf collection) and pUG6-32 (derived from pUG6, hph selection; [31]) using primers with homology to overhangs flanking the open reading frame of the particular gene according to [32]. Yeast cells were transformed as described in [33].

### 4.3. Staining of Cells by AlexaFluor488 5-TFP

Cells were vitally stained with AlexaFluor488 carboxylic acid, 2,3,5,6-tetrafluorophenyl ester amine-reactive probe (molecular probes). Labeling was performed according to the manufacturer’s manual. In brief, cells in 0.1 M sodium bicarbonate buffer, pH 8.6 were stained with the probe dissolved in DMSO (final concentration 0.1 mg/mL). After 20 min at room temperature, cells were washed 3 times with sterile water to remove the unbound probe. Then, 1 μL drops of stained cells in water were used for giant colony inoculation (cell concentration ~5 × 10^9^ cells/mL). 

### 4.4. Microscopy of Cells in Multicellular Structures

Analysis of cell growth on agar: Cells and microcolonies growing in the inoculated area were imaged by a Leica DMR microscope with a GFP filter and in the bright field, using NIS Elements software (Laboratory Imaging, Inc.).

Analysis of vertical colony cross-sections: the internal structure of chimeric giant colonies and biofilms was visualized by two photon excitation confocal microscopy as described [16,34]. In brief, colonies or biofilms were embedded in agarose and cut vertically with the cut surface placed on a coverslip. Side views of colonies were obtained by 2PE-CM using a confocal scanning microscope (SP8 AOBS VLL MP; Leica) fitted with a mode-locked laser (Ti:Sapphire Chameleon Ultra; Coherent Inc.) and 20×/0.70 or 63×/1.20 water immersion plan Apochromat objectives. Excitation wavelengths of 920 nm was used with the emission bandwidths of 480–595 nm and 600–740 nm for GFP and cell autofluorescence detection. Images of the whole colonies and the insets (Figure 1) were obtained by combining two or more images from neighboring fields of view. Alternatively, thin sections were prepared from giant colonies and biofilms and analyzed by fluorescence microscope as described [11]. In brief, colonies were embedded in 3% and biofilms in 4% agarose and sectioned using a Leica VT1200S vibrating microtome. Sections were observed using Carl Zeiss Axio Observer.Z1 fluorescence microscope equipped with an Axiocam 506 and an Apochromat 10×/0.45W objective or an Apochromat 63×/1.20W (for GFP expression or AlexaFluor488 5-TFP staining) using ZEN 2012 (blue edition) software. Filter sets for GFP (excitation 450–490 nm; emission 500–550 nm), for autofluorescence detection (excitation 538–562 nm; emission 570–640 nm), DIC, or bright field were used.

## Figures and Tables

**Table 1:** Strains used in this study.

Strain	Genotype	Source
BR-F	MATa/MATα, wild strain isolate	[15]
BR-F-p_TEF_-GFP	MATa/MATα, *his3*::NatMX-p_TEF_-GFP/*HIS3*	this study
BR-F-*rsr1*	MATa/MATα, *rsr1*Δ::KanMX/*rsr1*Δ:: HphMX	this study
BR-F-*rsr1*-p_TEF_-GFP	MATa/MATα, *rsr1*Δ::KanMX/*rsr1*Δ:: HphMX, *his3*::NatMX-p_TEF_-GFP/*HIS3*	this study
BR-F-*flo11*	MATa/MATα, *flo11*Δ::KanMX/*flo11*Δ::Ble	[16]
BR-F- *flo11*-p_TEF_-GFP	MATa/MATα *flo11*Δ::KanMX/*flo11*Δ:: Ble, *his3*::HphMX-p_TEF_-GFP/*HIS3*	J. Maršíková
BY4742	MATα, *his3*Δ1, *leu2*Δ0, *lys2*Δ0, *ura3*Δ0	Euroscarf
BY-p_TEF_-GFP	MATα, *leu2*Δ0, *lys2*Δ0, *ura3*Δ0, *his3*::NatMX-p_TEF_-GFP	[12]
BY-*ace2*	MATα, *his3*Δ1, *leu2*Δ0, *lys2*Δ0, *ura3*Δ0, *ace2*::KanMX	this study
BY-*ace2* -p_TEF_-GFP	MATα, *leu2*Δ0, *lys2*Δ0, *ura3*Δ0, *ace2*::KanMX, *his3*::NatMX-p_TEF_-GFP	this study
BY-*rsr1*	MATα, *his3*Δ1, *leu2*Δ0, *lys2*Δ0, *ura3*Δ0, *rsr1*::KanMX	this study
BY-*rsr1*-p_TEF_-GFP	MATα, *leu2*Δ0, *lys2*Δ0, *ura3*Δ0, *rsr1*::KanMX, *his3*::NatMX-p_TEF_-GFP	this study
BY-*bud2*	MATα, *his3*Δ1, *leu2*Δ0, *lys2*Δ0, *ura3*Δ0, *bud2*::KanMX	this study
BY-*bud2* -p_TEF_-GFP	MATα, *leu2*Δ0, *lys2*Δ0, *ura3*Δ0, *bud2*::KanMX, *his3*::NatMX-p_TEF_-GFP	this study

## Abbreviations

3D	Three-dimensional
2PE-CM	two photon confocal microscopy
ECM	extracellular matrix
AlexaFluor488 5-TFP	AlexaFluor488 carboxylic acid, 2,3,5,6-tetrafluorophenyl ester amine-reactive probe
DIC	differential interference contrast
